# American Society of Anesthesiologists Physical Status Classification: Hybrid Machine Learning–Large Language Model Ensemble Retrospective Validation Study

**DOI:** 10.2196/90724

**Published:** 2026-11-05

**Authors:** Christabelle Pabalan, Alvin Stewart, Gabrielle Fisher, Andrew Fisher

**Affiliations:** 1 Qventus Inc, Mountain View, CA, United States; 2 Department of Anesthesiology, University of Arkansas Medical Center, Little Rock, AR, United States; 3 Department Anesthesiology and Perioperative Medicine, Medical University of South Carolina, 135 Rutledge Ave, Charleston, SC, 29425, United States, +1 (843) 792-2322

**Keywords:** machine learning, perioperative care, perioperative risk stratification, large language models, American Society of Anesthesiologists Physical Status classification, ASA-PS, ASA physical status classification

## Abstract

**Background:**

The American Society of Anesthesiologists Physical Status (ASA-PS) classification system fundamentally shapes perioperative care delivery but suffers from poor interrater reliability (0.4‐0.6). Machine learning (ML) models and large language models (LLMs) offer complementary remedies: ML processes structured data consistently but cannot interpret the nuance of clinical narratives, while LLMs parse narrative text and generate explanations but adhere inconsistently to diagnostic criteria.

**Objective:**

This study aimed to develop and evaluate a parallel ML-LLM ensemble that combines the complementary strengths of both approaches for automated ASA-PS classification.

**Methods:**

We retrospectively analyzed 3000 adult surgical encounters from the University of Arkansas for Medical Sciences (UAMS) between August 2024 and May 2025. Cases were randomly allocated to training (n=2000), validation (N=500), and held-out test sets (N=500). The validation set was used exclusively for architecture comparison and hyperparameter tuning, and the held-out test set was reserved for final model performance validation. We developed multiple architectures, including traditional ML models (Extreme Gradient Boosting [XGBoost], Light Gradient Boosting Machine [LightGBM], ExtraTrees), an LLM-only baseline (Generative Pretrained Transformer-4o [GPT-4o]), and hybrid approaches. The parallel ensemble processed structured data through XGBoost and unstructured clinical notes through GPT-4o independently, with outputs combined via weighted averaging. Model performance was evaluated using macro-*F*_1_-score, exact match accuracy, and ±1-class accuracy.

**Results:**

A theoretical performance ceiling of macro-*F*_1_-score=0.59 was determined through evaluation by an expert panel of 2 board-certified anesthesiologists (AF and GF) who independently rated 50 patient charts for ASA-PS scores. On the true held-out test set (N=500), the parallel ensemble (*α*=0.30) achieved a macro-*F*_1_-score of 0.530 (95% CI 0.350‐0.600), exact match accuracy of 0.630 (95% CI 0.580‐0.670), and ±1-class accuracy of 0.980 (95% CI 0.968‐0.992), exceeding the frozen LLM-only baseline evaluated on the same set (macro-*F*_1_-score 0.493, exact match accuracy 0.621,±1-class accuracy 0.981). On the validation set used for architecture selection, the ensemble outperformed structured-only ML (XGBoost macro-*F*_1_-score=0.333, *P*<.001), and the LLM-only model reached a macro-*F*_1_-score of 0.642 (95% CI 0.525‐0.691), nominally exceeding the expert panel ceiling. Per-class analysis attributed this to a perfect score on the 2 validation ASA-PS 5 cases. Excluding that class, the adjusted macro-*F*_1_-score was approximately 0.55, within the range of the parallel ensemble. The LLM component also generated explanations detailing comorbidities, severity descriptors, and functional status indicators.

**Conclusions:**

The parallel ML-LLM ensemble achieved performance approaching the theoretical ceiling established by the expert panel benchmark while providing interpretable clinical explanations. The ±1-class accuracy of 0.980 (95% CI 0.968‐0.992) may enhance operational safety by minimizing extreme misclassifications. This approach demonstrates how complementary AI architectures can enhance perioperative risk assessment, which is particularly valuable given current health care workforce shortages.

## Introduction

The American Society of Anesthesiologists Physical Status (ASA-PS) classification system fundamentally shapes perioperative care delivery. Since 1941 [1], this 6-tier stratification system has determined surgical venue selection [2], anesthesia staffing decisions [3], preoperative testing protocols, and reimbursement structures [4]. Every surgical patient receives an ASA-PS assignment, making it one of the most frequently used risk assessment tools in medicine, with millions of classifications performed daily worldwide.

Despite its central role, interpretation of the ASA-PS system by anesthesia providers is highly variable. Multiple studies document that 2 anesthesiologists evaluating the same patient agree on the exact ASA-PS class only 40%‐60% of the time [5-9]. This variability stems not from clinical inexperience but from fundamental differences in how providers interpret ASA-PS criteria. Such discordance creates downstream consequences: misclassification drives inappropriate resource usage, triggering unnecessary preoperative testing in low-risk patients while potentially missing optimization opportunities in high-risk patients.

The shift of ASA-PS classification from subjective assessment to standardized prediction offers potential benefits throughout the perioperative continuum. When patients’ charts automatically populate with suggested ASA-PS scores and supporting explanations, preoperative clinic staff can triage cases before practitioner review. For example, medically straightforward cases can be directed to telephone screening while referring complex cases to in-person evaluation. This early stratification reduces bottlenecks and improves resource allocation. Nursing staff can focus their expertise on optimization planning rather than initial risk assessment, while day-of-surgery anesthesiologists receive consistent evaluations that reduce duplicative chart review.

Recognizing these limitations, researchers have pursued computational solutions with mixed results. Machine learning (ML) approaches using random forests [10], support vector machines [11], and Extreme Gradient Boosting (XGBoost) [11,12] have achieved widely varying performance, with reported agreement rates ranging from 57% to 90% [13,14]. Wongtangman et al [13], for example, applied XGBoost to 35 preoperatively available variables from over 400,000 patients, achieving 57.2% exact match accuracy, while Ferrari et al [12] reported 90.7% concordance between gradient-boosting predictions and anesthesiologist assignments in pediatric cases. The prospective design of Ferrari et al [12] study displayed model predictions to day-of-surgery anesthesiologists before final ASA-PS assignment, potentially biasing providers toward algorithmic suggestions rather than measuring true independent agreement. More recently, large language models (LLMs) like GPT-4 [15] and BioClinicalBERT (Massachusetts Institute of Technology; Computer Science and Artificial Intelligence Laboratory) [16] demonstrated impressive performance on sets of clinical notes and records, with some metrics exceeding human performance (accuracy of 69.6 ± 1.0% in Lew et al [14], *F*_1_-score of 0.50 in Chung et al [15]).

These computational approaches reveal a fundamental trade-off. ML models excel at consistently processing structured data but cannot interpret the clinical narratives that often contain critical nuance. Distinguishing “well-controlled” from “poorly-controlled” disease requires understanding beyond laboratory values and is often discussed within the text of a patient’s clinical notes. Conversely, LLMs demonstrate an ability to parse clinical language but show inconsistent adherence to diagnostic criteria and experience performance degradation with real-world data complexity [17].

Previous attempts to address these limitations through model combination have followed predictable patterns. Researchers have stacked homogeneous models: pure ML ensembles using multiple gradient boosting models [18], pure LLM ensembles combining language models through voting [19], and sequential hybrids using deep learning feature extraction followed by traditional ML [20]. Others have applied hybrid ML-LLM frameworks in clinical prediction tasks [21,22]. However, these sequential architectures introduce anchoring bias, where initial predictions influence subsequent models, potentially compromising overall performance.

We present a parallel ML-LLM ensemble designed to leverage the strengths of both approaches while mitigating their individual weaknesses. By processing structured data through XGBoost and clinical narratives through Generative Pretrained Transformer-4o (GPT-4o) independently before integration, we avoid sequential dependencies and potential anchoring effects. Here, we evaluate whether this architecture meaningfully improves ASA-PS prediction reliability and explore the potential utility of generated explanations.

## Methods

### Recruitment and Eligibility

This study is reported in accordance with the Transparent Reporting of a Multivariable Prediction Model for Individual Prognosis or Diagnosis-AI (TRIPOD-AI) guidelines. The study included adult patients (≥18 years) who were scheduled for surgical procedures between August 26, 2024, and May 5, 2025, across a range of surgical specialties at UAMS (Textbox 1).

Inclusion criteria included a documented ASA-PS score assigned by the attending anesthesiologist, at least 1 preoperative clinical note dated prior to the date of procedure, and structured clinical data with less than 95% missingness. Exclusion criteria included absence of any preoperative clinical notes, missing ASA-PS scores, structured data missingness greater than 95%, emergency cases, and canceled procedures.

Textbox 1. **Surgical services within the surgical encounters included. Distribution of surgical specialties represented in the 3,000 adult surgical encounters analyzed between August 26, 2024, and May 5, 2025.**

- Specialty
- Cardiology (Medical)
- Cardiothoracic Surgery
- Gastroenterology
- General Surgery
- Gynecology (Surgical)
- Neurosurgery
- Obstetrics
- Ophthalmology
- Orthopedic Surgery
- Orthopedic Surgery-Joints
- Orthopedic Surgery-Trauma
- Otolaryngology (Ear, Nose, and Throat)
- Pain Management (Interventional)
- Plastic Surgery
- Pulmonary (Bronchoscopy)
- Transplant Surgery
- Urogynecology
- Urology
- Vascular Surgery

### Dataset Composition

From the total cohort meeting eligibility criteria, 3000 encounters were included. Demographic features between the training, validation, and testing cohorts were compared to ensure the data split did not introduce confounding imbalances (Table 1). ASA-PS 5 cases, representing moribund patients, are rarely encountered in elective preoperative settings and were correspondingly rare here; their scarcity renders class-level estimates unstable at this sample size and limits their relevance to the elective use case.

Age was compared using an independent 2-sample *t* test. Gender and language were assessed using chi-square tests. Race and ethnicity data were not available and therefore could not be evaluated. No prior sample size or power calculation was performed. Instead, the study included all eligible cases within the retrospective study period (between August 26, 2024, and May 5, 2025).

**Table 1.** Patient demographics^a^.

Feature	Training set (n=2000)	Validation set (n=500)	True test set (n=500)	*P* values (Train vs test)	*P* values (Validation vs test)
Age	55.9 (17.8)^b^	56.7 (16.8)^b^	56.5 (17.3)^b^	.46	.89
Female	1063 (53.1%)^c^	276 (55.2%)^c^	264 (52.8%)^c^	.93	.49
English primary language	1916 (95.8%)^c^	486 (97.2%)^c^	482 (96.4%)^c^	.63	.59

a Statistical comparisons performed using independent 2-sample *t*-test for continuous variables (age) and chi-square test for categorical variables (gender, language).

b Mean (SD).

c n (%).

### Data Preprocessing

Only information documented prior to the scheduled surgical start time was used as model input to best approximate the ASA-PS score assigned by a day-of-surgery anesthesiologist. Structured data comprised more than 400 preoperative variables, including demographics, comorbidities, laboratory values, vital signs, medications, and procedural metadata. Language and gender were excluded from model inputs due to the risk that their inclusion could encode systemic documentation inequities and rater bias into ASA-PS predictions rather than true clinical signal [23-26]. Age was retained as a clinically validated predictor of comorbidity burden and perioperative risk, consistent with its established role across surgical risk stratification literature [27]. Incarceration status was retained under the same rationale. Unlike gender, language, race, and ethnicity, whose associations with assigned ASA-PS plausibly reflect documentation practices and rater perception rather than incremental clinical information, incarceration is associated with systematically elevated burdens of chronic, infectious, psychiatric, and substance use disease and with disrupted access to longitudinal care that are incompletely captured by structured comorbidity fields [28,29]. It was therefore treated as a marker of clinical risk exposure, analogous to age, rather than as a demographic descriptor. Requests for structured data components can be directed to the corresponding author (AF). Across the 3000 included encounters, median structured data missingness was 57.6% (IQR 38.8%‐92.5%). This sparsity primarily reflects the wide-pivoted feature representation, in which each column encodes a specific clinical concept and disease-specific variables are legitimately null for lower-acuity patients, rather than data-quality failure. Numeric missingness was mean-imputed using an imputer fit on the training data, while categorical missingness was retained as an explicit level; a stricter missingness threshold would have preferentially excluded the lower-acuity majority.

Medical diagnoses were captured through 2 complementary approaches: (1) direct extraction from electronic health record problem lists and *International Statistical Classification of Diseases, *Tenth *Revision* (*ICD-10*) codes, and (2) a proprietary LLM pipeline that analyzed free-text notes to identify additional medical diagnoses not recorded in structured fields. Unstructured notes were filtered to 50 curated note types, including progress notes, history and physical, discharge summaries, operative notes, anesthesia evaluations, emergency department notes, consult notes, and nursing notes. Duplicates were deduplicated on patient ID and note ID, keeping the most recent version. All notes per patient were concatenated and truncated to 100,000 tokens, preserving the latest content. A 3-year lookback window was applied. No further text cleaning was performed; raw plain text was preserved to retain clinical nuance.

### Training and Test Set Split

From the total cohort of 3000 encounters, cases were randomly allocated to a training set (n=2000), a validation set (n=500), and a held-out test set (n=500). Cases were assigned to training, validation, and test sets using stratified random sampling based on documented ASA-PS scores, to ensure balanced representation of ASA-PS classes across all 3 sets.

### Validation Protocol

For all model training and final testing, the day-of-procedure anesthesiologist-assigned ASA-PS served as the ground truth. Each case reflects the assessment of a single attending anesthesiologist as documented in the electronic health record; no multirater adjudication or consensus process was applied to the reference labels. This approach was selected to follow established literature [11-14,16] for automated ASA-PS classification predictions and to ensure the model is assessed against the real-world clinical benchmark, including the inherent label uncertainty introduced by known interrater variability.

Three distinct LLM prompts were used, each serving a separate functional role: (1) clinical diagnosis extraction from unstructured notes, (2) ASA-PS classification, and (3) generation of structured ASA-PS explanations for a subset of examined architectures. All 3 were implemented using GPT-4o queried at temperature=0 to ensure deterministic outputs, with concatenated patient notes truncated to 100,000 tokens prior to submission and responses returned in structured JSON format.

The clinical diagnosis extraction prompt analyzed free-text notes to identify medical diagnoses not recorded in structured electronic health record fields, supplementing the problem-list and *ICD-10* data described above. Extraction performance was validated in a sample of 221 UAMS patients, separate from the 3,000-encounter model development cohort. For each patient, a predefined set of 168 candidate clinical facts was evaluated, yielding 37,128 patient-fact units. The reference standard was established by 2 board-certified anesthesiologists, who each reviewed the anesthesia preprocedure evaluation for all 221 patients and determined which of the 168 facts were captured in that documentation. The extraction pipeline is internally validated at a sensitivity of 0.738 (95% CI 0.719‐0.752), precision of 0.845 (95% CI 0.832‐0.857), and specificity of 0.978 (95% CI 0.976‐0.980), and its outputs were folded into the structured feature set supplied to the ML models. CIs were estimated by bootstrap resampling at the patient level to account for the nonindependence of facts within a patient.

The ASA-PS classification prompt was used to generate the ASA-PS prediction in the LLM-only baseline and as the LLM stream within the hybrid architectures. This prompt was structured around 3 sequential modules: clinical information extraction, severity grading, and ASA-PS class assignment. The model was instructed to reason through each module before producing a final output, encouraging consistent intermediate reasoning analogous to the regularization effect of multitask learning. It was anchored to the ASA 2020 Physical Status criteria, which informed the threshold-based decision logic embedded in the severity-grading module, so that the model performed criteria-anchored reasoning rather than relying on pretraining exposure to ASA-PS language alone. Each response contained a final ASA-PS prediction, binary indicators for ASA-PS classes 1-5, a brief clinical rationale, and a confidence score; because temperature was fixed at 0, top-p (nucleus) sampling was nonoperative, and max_tokens was left unconstrained (responses averaged well under 500 tokens). The complete template, including its scaffolded reasoning structure and embedded decision logic, is available to qualified investigators upon request to the corresponding author AF.

A third ASA-PS explanation prompt was used in the Sequential Hybrid (with and without embeddings) and Parallel Ensemble architectures. This prompt generated structured explanations of each score, outlining the key comorbidities, severity descriptors, and functional-status indicators that contributed to it. These explanations were constrained to a standardized format to enable consistent interpretation and independent adjudication of the model’s reasoning. Because XGBoost is the terminal step in the LLM-as-Features alternative, no LLM-guided explanation was generated in that pipeline.

The XGBoost, Light Gradient Boosting Machine (LightGBM), and ExtraTrees models were trained using a standardized pipeline with stratified 3-fold cross-validation and Optuna-based (Bayesian) hyperparameter optimization. A shared preprocessing pipeline was applied to the structured feature set before model fitting, so that all 3 classifiers received identical inputs. Features with ≥95% missingness were dropped. For categorical features, missing values were retained as an explicit “missing” level prior to label encoding, preserving nonrecording as potentially informative. Remaining numeric features were mean-imputed using an imputer fit on the training data alone and applied unchanged to the validation and held-out test sets, preventing test-set information from entering the imputation statistics. Random oversampling of minority ASA-PS classes was then applied to the training data only, after imputation, so that no imputation statistic was computed from duplicated rows. Validation and held-out test sets retained their native class distributions. The objective was to maximize macro-averaged *F*_1_-score across ASA-PS classes, with stratified folds preserving the class distribution within each cross-validation split. For each model, the dataset was split into 3 stratified folds, with Optuna tuning performed within each fold; key hyperparameters (eg, tree depth, learning rate, and regularization strength) were searched within predefined ranges. The mean macro-*F*_1_-score across folds was used to select optimal hyperparameters, and final models were retrained on the full training set and evaluated on the validation set. All stochastic operations (cross-validation splits, Optuna sampling, and final model training) used a fixed random seed (42) to ensure reproducibility. An LLM-only baseline used GPT-4o to predict ASA-PS scores from preoperative notes alone, applying the ASA-PS classification prompt described above. Notes-only input consistently outperformed configurations that added structured data, so the final pipeline used text alone.

Hybrid architecture development is depicted in Figure 1.

**Figure 1.**
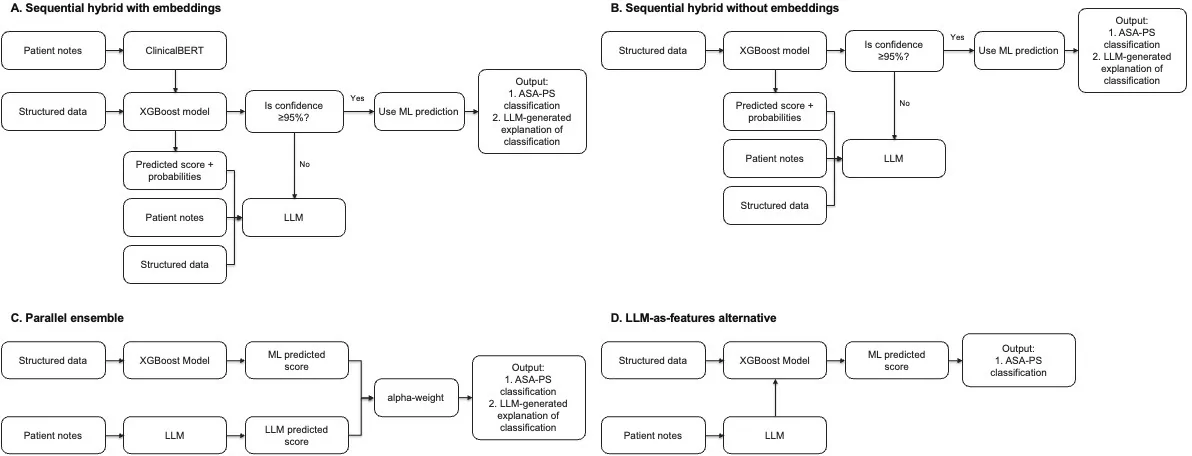
Schematic diagrams of the 4 candidate ML and LLM hybrid architectures evaluated for automated ASA-PS classification. ASA-PS: American Society of Anesthesiologists-Physical Status; ClinicalBERT: Clinical Bidirectional Encoder Representations from Transformers; GPT: Generative Pretrained Transformer; LLM: large language model; ML: machine learning; XGBoost: Extreme Gradient Boosting.

In the Sequential Hybrid with Embeddings architecture (Figure 1A), structured data were combined with 768-dimensional Clinical Bidirectional Encoder Representations from Transformers (ClinicalBERT) embeddings of patient notes to create an augmented feature vector, which was used to train an XGBoost classifier. A confidence gating mechanism routed high-confidence cases (≥95%) to XGBoost directly, while lower-confidence cases were forwarded to GPT-4o for final classification with access to notes, structured data, and the ML prediction (Figure 1). Performance was assessed using macro-*F*_1_-score and accuracy, with coverage analysis to quantify the proportion of cases handled by ML versus LLM. All evaluations were conducted on the validation set.

In the Sequential Hybrid without Embeddings architecture (Figure 1B), structured data were used to train an XGBoost classifier, with a confidence gating mechanism routing high-confidence cases (≥95%) to XGBoost and low-confidence cases to GPT-4o. GPT-4o received the original notes, structured data summary, and ML prediction. Performance was assessed using the same metrics as the embedding pipeline to isolate the effect of excluding ClinicalBERT embeddings, with additional analysis of confidence coverage and anchoring bias. XGBoost output probabilities were not formally calibrated (eg, via Isotonic Regression or Platt Scaling) prior to applying the confidence gating threshold. While preliminary internal review suggested calibration did not substantially alter routing behavior or downstream performance, formal prospective calibration using out-of-fold training predictions is recommended before clinical deployment.

In the Parallel Ensemble architecture (Figure 1C), structured data were processed by XGBoost and clinical notes by GPT-4o in fully independent streams to avoid the anchoring effects of sequential designs. Final predictions were generated through weighted averaging of the ML probability distribution and LLM classification (optimal *α*=.30, corresponding to 30% ML and 70% LLM). Performance was evaluated against individual components using macro-*F*_1_, accuracy, and calibration metrics, with sensitivity analysis across weighting values.

In the LLM-as-Features Alternative (Figure 1D), GPT-4o was first prompted to generate ASA-PS predictions and intermediate markers (binary flags for ASA-PS classes 1-5) from clinical notes. These outputs were concatenated with the 400+ structured clinical variables to form an augmented feature set, which was used to train a second XGBoost model. This allowed the algorithm to learn optimal weighting between structured data and LLM-derived features. The same structured feature set and the same preprocessing pipeline described above were used across all hybrid model architectures; no features were added or removed between the sequential and parallel configurations. Structured variables were drawn from 8 source categories: chart-mined clinical facts, *ICD-10* condition matrices, case-level features (eg, surgical urgency and procedure risk), 34 laboratory value types, 9 vital sign types, device flags, medication flags, and age.

Feature importance was quantified via Shapley additive explanations (SHAP) TreeExplainer on the XGBoost component of the parallel ensemble. Per-class SHAP summary plots, showing the direction of each feature’s contribution to every ASA-PS class, are provided in Multimedia Appendix 1.

Model performance was evaluated using macro-averaged *F*_1_, exact-match accuracy, and ±1-class accuracy. Ninety-five percent CIs were estimated by bootstrap resampling (1000 iterations), and pairwise model comparisons used permutation testing (1000 permutations), with *P*<.05 considered statistically significant. Analyses were performed in Python (Python Software Foundation, Wilmington, DE, United States); package versions for XGBoost, LightGBM, scikit-learn, Optuna, and SHAP, along with the exact GPT-4o snapshot and the ClinicalBERT variant, were used for the 768-dimensional embeddings.

To quantify the inherent variability in provider interpretation of the ASA-PS classification system and establish a local performance ceiling, we conducted an internal reliability study independent of all automated pipelines. Two board-certified anesthesiologists (AF, GF) independently reviewed a subset of 50 randomly selected cases drawn from the broader eligible cohort, separate from the validation and held-out test sets, and assigned ASA-PS scores based solely on preoperative documentation. Agreement for each pairwise comparison was quantified as macro-*F*_1_.

Both reviewers maintain active clinical responsibilities that include preoperative patient management and are external to the source institution, providing independent perspective and guarding against local anchoring bias. Because these cases were sequestered from all model development and evaluation sets, the resulting ceiling did not influence hyperparameter tuning, architecture selection, or alpha weighting.

The expert ceiling was computed from 3 rating sources for each of the 50 cases: the 2 panel anesthesiologists and the day-of-surgery attending anesthesiologist whose documented ASA-PS served as the reference standard throughout this study. Three pairwise comparisons are defined among these 3 sources: panel anesthesiologist 1 versus the day-of-surgery attending, panel anesthesiologist 2 versus the day-of-surgery attending, and panel anesthesiologist 1 versus panel anesthesiologist 2. The ceiling of 0.59 is the mean macro-*F*_1_-score across these 3 comparisons. Macro-*F*_1_-score is symmetric under exchange of the 2 rating sets, so the panel-versus-panel comparison does not require designating either rater as the reference. Each comparison was computed on the cases for which both members of the pair provided a rating. This mean reflects both alignment with the clinical reference standard and interreviewer concordance, and is consistent with prior literature demonstrating moderate interrater reliability for ASA-PS scoring (κ ≈ 0.4‐0.6), where perfect alignment is neither clinically expected nor mathematically attainable. Three rating sources across 50 cases yield 150 possible ratings, of which 137 were available: all 50 day-of-surgery ratings, present by inclusion criteria, and 87 of 100 possible panel ratings, with 13 omitted because insufficient documentation precluded expert classification. The per-class distribution of all 137 ratings was ASA-PS 1 (n=17), ASA-PS 2 (n=31), ASA-PS 3 (n=52), ASA-PS 4 (n=33), and ASA-PS 5 (n=4).

### Ethical Considerations

This retrospective observational study was approved by the Institutional Review Board (IRB) of the University of Arkansas for Medical Sciences (UAMS). Given the retrospective nature of the study and use of existing clinical data, the IRB granted a waiver of informed consent (IRB number 298590); the original clinical data were collected under standard institutional consent practices that permit secondary analysis. Direct identifiers were not retained in the analytic dataset, and all coded data were stored in a secure, Health Insurance Portability and Accountability Act (HIPAA)-compliant Amazon Web Services environment maintained by Qventus under a Business Associate Agreement. No compensation was provided to study participants. No identifiable patient images are included in this paper or Multimedia Appendix 1.

## Results

All architecture comparisons were conducted on the validation set (N=500); true held-out test set results for selected architectures are reported separately below.

The XGBoost model achieved a macro-*F*_1_-score of 0.333 (95% CI 0.288‐0.388), exact match accuracy of 0.662 (95% CI 0.622‐0.706), and exact match ±1-class (ie, within 1 ASA-PS level of predicted) accuracy of 0.980 (95% CI 0.968‐0.992). The LightGBM model achieved a macro-*F*_1_-score of 0.321 (95% CI 0.270‐0.422), exact match accuracy of 0.662 (95% CI 0.624‐0.704), and exact match ±1-class accuracy of 0.988 (95% CI 0.978‐0.996). ExtraTrees achieved a macro-*F*_1_-score of 0.303 (95% CI 0.261‐0.404), exact match accuracy of 0.600 (95% CI 0.556‐0.648), and exact match ±1-class accuracy of 0.958 (95% CI 0.942‐0.976; Table 2). At this sample size, macro-*F*_1_-score should be interpreted in conjunction with bootstrap CI and complementary metrics rather than as a standalone point estimate.

**Table 2.** Model performance on the validation set (N=500) by macro-*F*_1_-score, exact match accuracy, and exact match ±1-class accuracy^a^.

Model	Macro-*F*_1_ [95% CI]	Exact match^b^ [95% CI]	±1-Class^b^ [95% CI]
Traditional ML^c^			
XGBoost^d^	0.333 [0.288‐0.388]	0.662 [0.622‐0.706]	0.980 [0.968‐0.992]
LightGBM^e^	0.321 [0.270‐0.422]	0.662 [0.624‐0.704]	0.988 [0.978‐0.996]
ExtraTrees	0.303 [0.261‐0.404]	0.600 [0.556‐0.648]	0.958 [0.942‐0.976]
LLM Only	0.642 [0.525‐0.691]	0.668 [0.628‐0.708]	0.982 [0.970‐0.992]
Hybrid architecture models			
Sequential Hybrid with Embeddings	0.463 [0.283‐0.569]	0.626 [0.582‐0.666]	0.978 [0.964‐0.990]
Sequential Hybrid without Embeddings	0.475 [0.298‐0.582]	0.634 [0.594‐0.674]	0.982 [0.970‐0.992]
LLM-as-Features pipeline	0.542 [0.480‐0.598]	0.670 [0.630‐0.710]	0.990 [0.982‐0.998]
Parallel Ensemble	0.574 [0.404‐0.671]	0.666 [0.622‐0.710]	0.982 [0.970‐0.992]

a Performance comparison of traditional ML models (XGBoost, LightGBM, ExtraTrees), a standalone LLM (GPT-4o), and 4 hybrid architectures on the validation set (N=500). All architecture comparisons used this set; the held-out test set was reserved for final evaluation of the selected model and was not used during model development or hyperparameter selection. Metrics include macro-*F*_1_-score (balanced performance across all American Society of Anesthesiologists-Physical Status classes), exact match accuracy (percentage of predictions matching anesthesiologist-assigned scores), and ±1-class accuracy (percentage within the American Society of Anesthesiologists-Physical Status class of the reference standard). Bootstrap CIs were computed over 1000 resamples.

b Accuracy.

c ML: machine learning.

d XGBoost: Extreme Gradient Boosting.

e LightGBM: Light Gradient Boosting Machine.

The LLM-Only model achieved a macro-*F*_1_-score of 0.642 (95% CI 0.525‐0.691), exact match accuracy of 0.668 (95% CI 0.628‐0.708), and exact match ±1-class accuracy of 0.982 (95% CI 0.970‐0.992; Table 2).

The Sequential Hybrid with Embeddings model achieved a macro-*F*_1_-score of 0.463 (95% CI 0.283‐0.569), exact match accuracy of 0.626 (95% CI 0.582‐0.666), and exact match ±1-class accuracy of 0.978 (95% CI 0.964‐0.990). Within this model, 131 cases were handled by ML, and 369 were routed to LLM.

The Sequential Hybrid without Embeddings model achieved a macro-*F*_1_-score of 0.475 (95% CI 0.298‐0.582), exact match accuracy of 0.634 (95% CI 0.594‐0.674), and exact match ±1-class accuracy of 0.982 (95% CI 0.970‐0.992). Within this model, 174 cases were handled by ML, and 326 were routed to LLM.

The LLM-as-Features Pipeline model achieved a macro-*F*_1_-score of 0.542 (95% CI 0.480‐0.598), exact match accuracy of 0.670 (95% CI 0.630‐0.710), and exact match ±1-class accuracy of 0.990 (95% CI 0.982‐0.998; Table 2).

Confusion matrices for all evaluated models are presented in Figure 2 and clarify the divergence between exact-match accuracy and macro-*F*_1_-score. The structured-only models concentrated predictions in the majority ASA-PS II and III classes, correctly classifying 78%‐85% of ASA-PS III cases but only 24%‐41% of ASA-PS I and none of the high-acuity classes: every ASA-PS V case and nearly every ASA-PS IV case was misclassified (XGBoost and LightGBM assigned all 29 ASA-PS IV cases to ASA-PS III; ExtraTrees recovered (2/29 cases). This majority-class collapse explains why structured-only exact-match accuracy (~0.66) coexists with low macro-*F*_1_-score (~0.30): accuracy is carried by the prevalent classes, whereas macro-*F*_1_-score exposes the failure on the rare ones. Incorporating GPT-4o’s clinical narrative restored minority-class recovery: the LLM-only model correctly classified 59% of ASA-PS I, 45% of ASA-PS IV, and both ASA-PS V cases (2/2), and the Parallel Ensemble matched this profile (ASA-PS I 59%, ASA-PS IV 45%) while correctly classifying 1 of 2 ASA-PS V cases. This recovery is the source of the higher macro-*F*_1_-score of the LLM-incorporating architectures, and it pinpoints the LLM-only model’s apparent excess over the 0.59 benchmark: with only 2 ASA-PS V cases, a perfect 2/2 mechanically inflates the unweighted macro-average, whereas the ensemble’s structured stream reassigned one (ASA-PS V recall 50%), yielding its more conservative macro-*F*_1_-score of 0.574.

**Figure 2.**
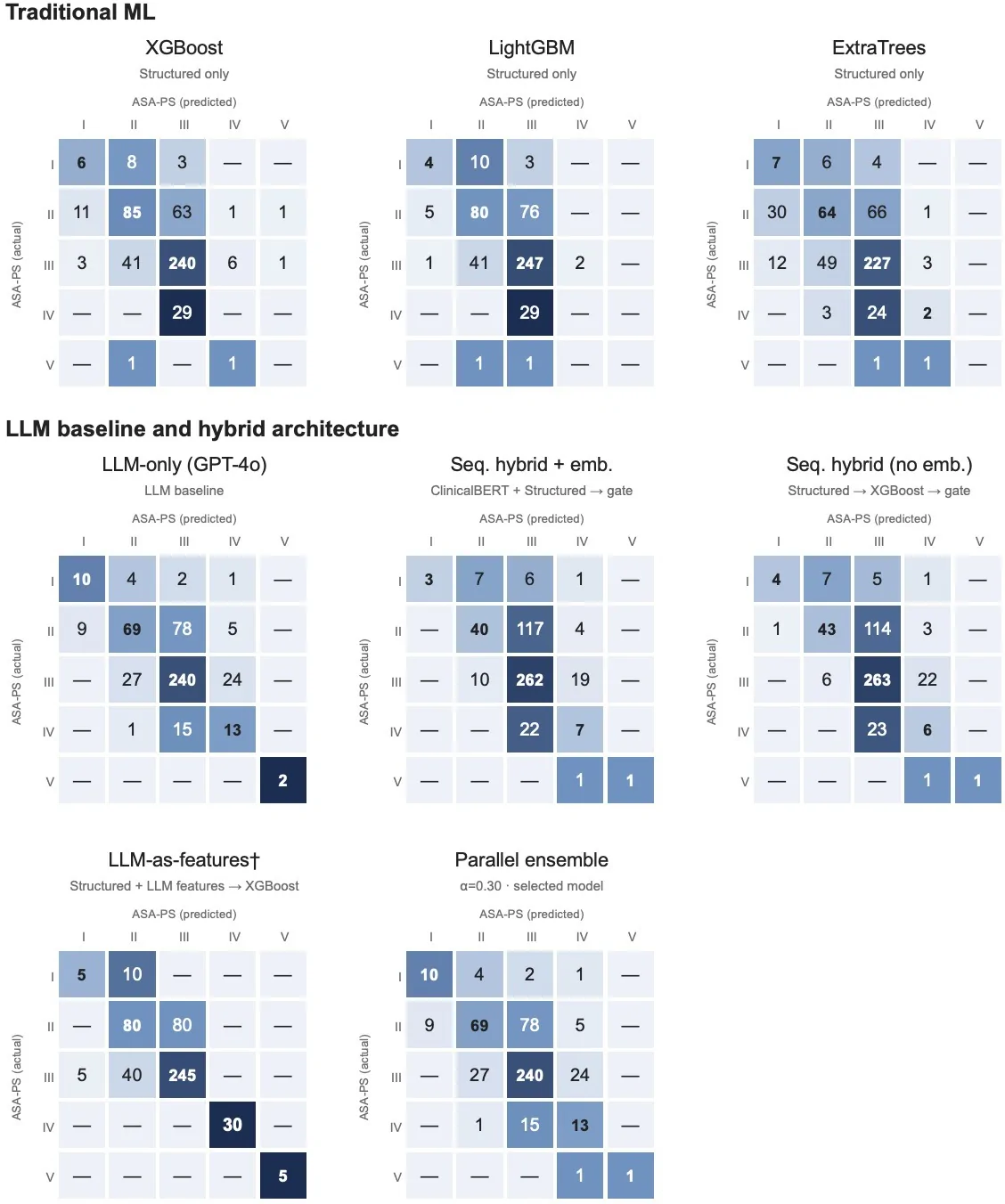
Confusion matrices comparing each model’s predicted ASA-PS class (columns) with the day-of-surgery, anesthesiologist-assigned reference class (rows) on the validation set (n=500; reference distribution ASA-PS I=17, II =161, III =291, IV =29, V=2). ASA-PS: American Society of Anesthesiologists-Physical Status; Emb.: embedding; GPT: Generative Pretrained Transformer; LightGBM: Light Gradient Boosting Machine; LLM: large language model; ML: machine learning; Seq.: sequential; XGBoost: Extreme Gradient Boosting.

To determine the optimal weighting between the ML model and the LLM in the Parallel Ensemble, we conducted a sweep across alpha values ranging from 1.0 (pure ML) to 0.0 (pure LLM, Figure 3; Full statistical table provided in Multimedia Appendix 1). At its selected weighting (*α*=.30), the Parallel Ensemble achieved a validation set macro-*F*_1_-score of 0.574 (95% CI 0.404‐0.671), exact-match accuracy of 0.666 (95% CI 0.622‐0.710), and ±1-class accuracy of 0.982 (95% CI 0.970‐0.992; Table 2). Although the validation macro-*F*_1_-score for the parallel ensemble (0.574) was nominally below the LLM-only baseline (0.642), the two proved statistically indistinguishable on the validation set.

**Figure 3.**
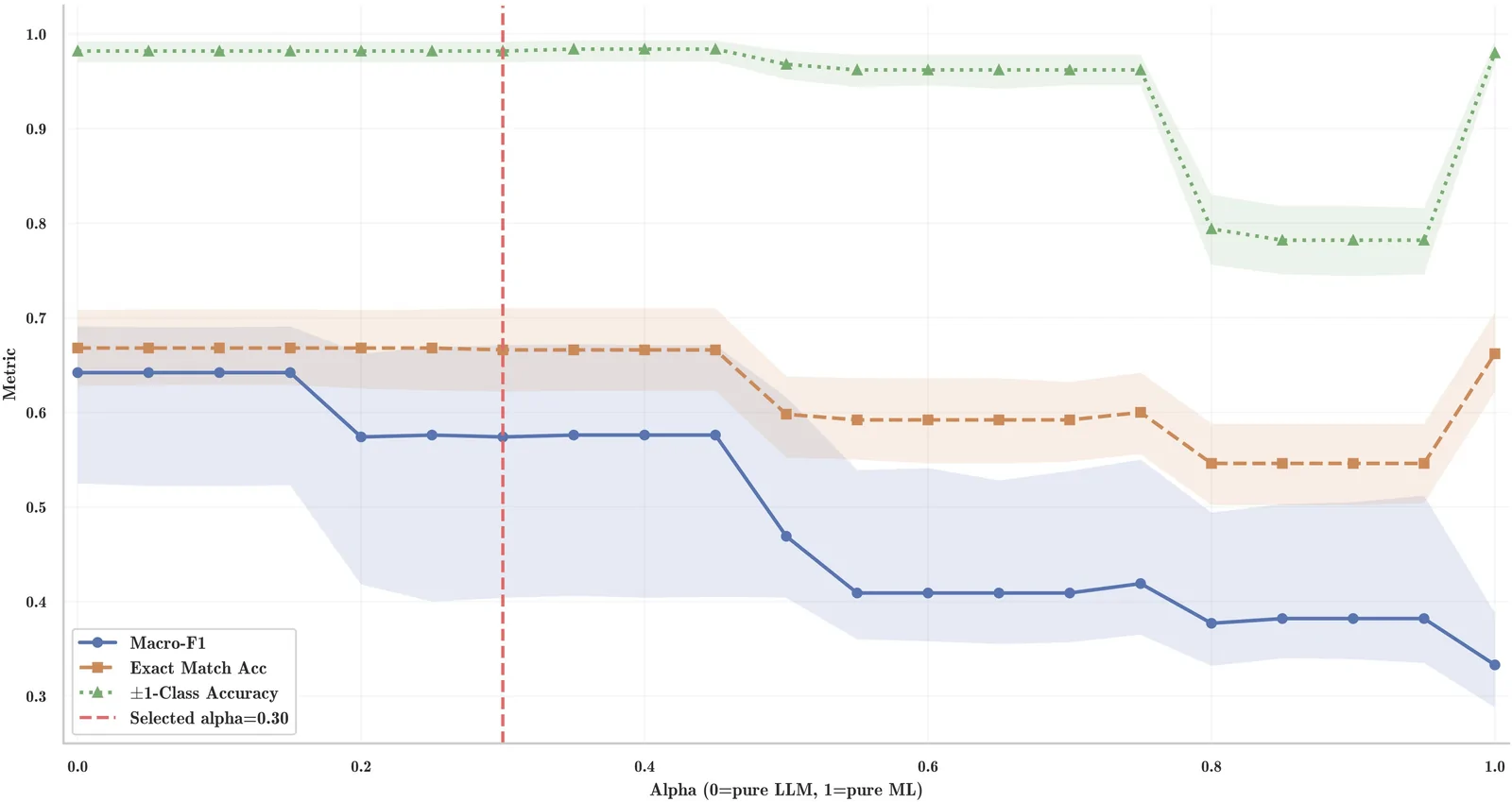
Macro-*F*_1_, exact match accuracy, and ±1-class accuracy across the full alpha sweep on the validation set (n=500) with 95% bootstrap CIs. Alpha was selected at *α*=.30, which falls within a stable region of equivalent macro-*F*_1_-score performance (α=.20‐.45), confirming the selection is not sensitive to the precise weighting value. This point represents the inflection between the LLM-dominant region (where the *F*_1_-score of 0.64 exceeds the expert panel benchmark of 0.59 (95% CI 0.44‐0.70), suggesting class-distribution sensitivity) and the ML-dominant region where *F*_1_-score degrades substantially. Macro-*F*_1_-score remains relatively stable between α=0.20 and α=0.45, supporting the selection of α=0.30. Performance generally declines at higher alpha values, with particularly pronounced reductions in ±1-class accuracy between α=0.80 and α=0.95. Although exact-match and ±1-class accuracy recover at α=1.00 (pure XGBoost), Macro-*F*_1_-score reaches its lowest value, highlighting the trade-offs between overall accuracy and class-balanced performance. Wide macro-*F*_1_-score CIs are consistent with bootstrap sensitivity to minority class distributions across all architectures at this sample size. LLM: large language model; ML: machine learning.

The mean macro-*F*_1_-score across the 3 prespecified pairwise comparisons (each panel expert versus the day-of-surgery reference standard, and the 2 panel experts compared directly) was 0.59 (95% CI 0.440‐0.700; bootstrap, 1000 iterations), establishing the study’s performance ceiling.

To confirm generalizability to a broader data set, the parallel ensemble was tested on a true held-out test set (N=500, unseen during all development decisions). The frozen model achieved a macro-*F*_1_-score of 0.530 (95% CI 0.350‐0.600), exact match accuracy of 0.630 (95% CI 0.580‐0.670), ±1-class accuracy of 0.980 (95% CI 0.968‐0.992), and Cohen κ=0.34. The modest generalization loss from validation (macro-*F*_1_-score=0.574) to held-out (0.530) is consistent with expected variance at this sample size and confirms that development-phase estimates were not substantially inflated by overfitting. SHAP values were computed by applying TreeExplainer to the finalized XGBoost model on the preprocessed validation feature matrix, using the same preprocessing pipeline fit on the training data and with no retraining on validation data; the mean absolute SHAP values reported in Table 3 are aggregated across all 5 ASA-PS classes.

**Table 3.** SHAP^a^ feature importance summary for the parallel ensemble XGBoost^b^ component^c^.

Feature	Mean SHAP	Minimum SHAP	Max SHAP	SHAP std
Age	0.602	0.118	1.422	0.170
Total medication count	0.468	0.089	1.981	0.294
Gastrointestinal hemorrhage	0.372	0.096	0.751	0.129
Pacemaker/AICD^d^	0.346	0.138	0.457	0.081
Vasopressin	0.341	0.165	0.782	0.101
Antianginal medication	0.340	0.122	0.612	0.098
Surgical urgency code	0.330	0.082	0.815	0.099
BMI	0.324	0.076	1.720	0.262
Renal failure	0.319	0.137	0.604	0.074
Hyperthyroidism	0.318	0.199	0.452	0.069

a SHAP: Shapley additive explanations.

b XGBoost: Extreme Gradient Boosting.

c Top clinical predictors ranked by mean absolute SHAP value from TreeExplainer analysis of the XGBoost component within the parallel ensemble (α=.30). Mean SHAP quantifies overall feature influence; directional SHAP (min/max) and standard deviation capture feature-level variability across the cohort. Age (0.602), total medication count (0.468), gastrointestinal hemorrhage (0.372), pacemaker/AICD status (0.346), vasopressin use (0.341), and surgical case urgency code (0.330) were the strongest contributors. All values are computed relative to the model baseline.

d AICD: automatic implantable cardioverter defibrillator.

The top predictors by mean absolute SHAP value were age (mean 0.602, SD 0.170), total medication count (mean 0.468, SD 0.294), gastrointestinal hemorrhage (mean 0.372, SD 0.129), pacemaker/automatic implantable cardioverter-defibrillator (mean 0.346, SD 0.081), vasopressin use (mean 0.341, SD 0.101), and surgical case urgency code (mean 0.330, SD 0.099).

Pairwise permutation testing (1000 permutations) confirmed that the parallel ensemble significantly outperformed the structured-only XGBoost baseline on validation macro-*F*_1_-score (*P*<.001). Differences versus sequential hybrid architectures were not statistically significant (*P*=.478-.482), attributable to limited power at N=500. The parallel ensemble and LLM-only baseline were statistically indistinguishable on both metrics (*P*>.99); model selection was therefore guided by architectural considerations and clinical plausibility, as described in the Discussion section.

For completeness of the ablation comparison, the LLM-only baseline was also evaluated on the held-out test set, achieving a macro-*F*_1_-score of 0.493 (95% CI 0.368‐0.585), exact match accuracy of 0.621 (95% CI 0.579‐0.662), and ±1-class accuracy of 0.981 (95% CI 0.969‐0.993). The parallel ensemble directionally outperformed the LLM-only baseline on macro-*F*_1_-score (0.530 vs 0.493), confirming the marginal contribution of the structured ML component. On the held-out test set, the LLM-only baseline (0.493) and parallel ensemble (0.530) both exceeded the structured-only XGBoost validation-set macro-*F*_1_-score (0.333; XGBoost was not separately evaluated on the held-out set), suggesting that each modality contributes incrementally to the final result.”

Structured explanations outlining key comorbidities, severity descriptors, and functional-status indicators contributing to the score were generated by the Sequential Hybrid with Embeddings, Sequential Hybrid without Embeddings, and Parallel Ensemble architectures. Deidentified sample outputs generated by the Parallel Ensemble architecture are denoted in Textbox 2.

Textbox 2. **Sample output for American Society of Anesthesiologists-Physical Status (ASA-PS) score prediction explanation for parallel ensemble pipeline. Sample explanatory outputs generated by the large language model (LLM) component of the parallel ensemble for each ASA-PS class (1-4).**

**ASA-PS classification prediction and LLM–generated explanation**

- 1: The patient is a 64-year-old female presenting with no significant medical history impacting her systemic health. Her medical notes indicate she is well-developed, well-nourished, and in no acute distress. She has a history of osteoporosis, but it is well-managed with supplements and does not impose functional limitations. Her review of systems is largely negative, with no significant chronic conditions or functional impairments noted. She is not on any complex medication regimen, and her daily activities are not restricted. Based on the absence of systemic disease and functional limitations, she fits the criteria for ASA 1, indicating a normal healthy patient.
- 2: The patient has a history of hypertension and diabetes mellitus (DM), both of which appear to be well-controlled. The blood glucose level of 110 mg/dL and blood pressure of 150/67 mm Hg, although slightly elevated, suggest that the conditions are managed with medication. There is no indication of severe systemic disease or substantial functional limitations. The patient is encouraged to exercise, indicating a mild systemic disease with minimal functional limitations. The fall and knee injury do not suggest a systemic disease but rather an isolated incident. Therefore, the patient’s condition aligns with ASA 2 criteria, indicating a mild systemic disease that is well-controlled.
- 3: The patient presents with a history of significant medical conditions, including diabetes mellitus, hypertension, and a past stroke. She has undergone multiple surgical interventions for airway management, including tracheostomy and bronchoscopy with dilation. Despite improvements, she still experiences functional limitations, such as difficulty with respirations during activity and a persistent tracheocutaneous fistula. Additionally, she is undergoing maintenance chemotherapy for breast cancer, indicating a complex medical regimen. These factors collectively suggest a severe systemic disease with substantial functional limitations, aligning with ASA 3 criteria.
- 4: The patient presents with a complex medical history that includes decompensated liver cirrhosis, chronic kidney disease stage 4, and recent simultaneous liver and kidney transplant. His condition is further complicated by ischemic cholangiopathy, requiring multiple ERCPs, and a recent episode of hematemesis with syncope. He is on a complex immunosuppressive regimen and has experienced significant functional limitations due to his medical conditions. The presence of life-threatening complications such as ischemic cholangiopathy and the need for constant medical intervention and monitoring indicate a constant threat to life, aligning with ASA 4 classification.

## Discussion

### Principal Findings

Among the evaluated configurations, the parallel ensemble (*α*=.30) was identified as the optimal architecture for ASA-PS classification. On the held-out test set, the parallel ensemble achieved a macro-*F*_1_-score of 0.530 (95% CI 0.350‐0.600), exact match accuracy of 0.630 (95% CI 0.580‐0.670), ±1-class accuracy of 0.980 (95% CI 0.968‐0.992), and Cohen κ of 0.34, confirming generalizability beyond the development cohort. On the validation set, the standalone LLM-only baseline achieved a macro-*F*_1_-score of 0.642 (95% CI 0.525‐0.691), nominally exceeding the expert panel ceiling of 0.59 (derived as the average macro-*F*_1_-score across 3 pairwise comparisons: each of 2 board-certified anesthesiologists compared against the day-of-surgery reference standard, and the 2 anesthesiologists compared with each other). This result requires careful interpretation, as it was not reproduced when the same frozen baseline was applied to the held-out test set, where the LLM-only architecture achieved a macro-*F*_1_-score of 0.493 (95% CI 0.368‐0.585). Per-class analysis localizes this discrepancy to a single class: ASA-PS V performance on the validation set (n=2, *F*_1_-score=1.00) mechanically inflates the unweighted macro average, a known artifact of macro-averaging under extreme class rarity, and this inflation is not reproduced on the held-out set. Excluding ASA-PS V, the remaining 4 validation classes yield a macro-*F*_1_-score of approximately 0.55, closely aligned with the parallel ensemble and within the expert interrater reference range. The held-out estimate of 0.493 is a substantially closer match to this ASA-PS V-excluded validation figure than to the nominal 0.642, and the held-out result should therefore be regarded as the more faithful representation of broader LLM-only performance. The apparent validation advantage reflects the arithmetic of 2 correctly classified ASA-PS V cases rather than a generalizable superiority over the expert ceiling.

LLM consistency and human validity are distinct properties: given documented interrater reliability of 0.4‐0.6 in ASA-PS classification, it is plausible that GPT-4o achieves greater internal consistency than individual raters. However, consistency with a human-labeled standard does not constitute clinical superiority, particularly when concentrated in 2 extreme-acuity cases. Convergence of LLM-only performance toward the parallel ensemble on the true test set further supports this interpretation. Pairwise permutation testing confirmed that the parallel ensemble and LLM-only baseline were statistically indistinguishable on macro-*F*_1_-score and exact match accuracy (*P*>.99). Model selection was therefore guided by architectural considerations: by independently integrating objective structured clinical data alongside LLM-derived narrative interpretation, the parallel ensemble anchors predictions to measurable clinical variables in a way an LLM-only approach cannot. This is particularly important for minority classes where structured indicators (eg, laboratory values, medication burden, and documented comorbidities) may diverge from narrative tone.

The test-set Cohen κ of 0.34 warrants direct consideration against the 0.4‐0.6 interrater reliability cited in the Introduction section. Published interrater coefficients are derived from controlled studies in which multiple raters score identical cases, whereas our kappa is computed against single-rater-day-of-surgery labels that already embed the full spectrum of interrater variability, so the model is evaluated against a moving target that human benchmarks never face. Kappa and macro-*F*_1_-score also capture different properties: kappa corrects for chance agreement and is sensitive to the ASA-PS II-III predominance of this cohort, mechanically depressing it relative to macro-*F*_1_-score. The case for deployment therefore rests not on matching expert kappa but on the 98.0% ±1-class accuracy, which captures the clinically operative distinction between acceptable and dangerous misclassification, together with the model’s capacity for consistent, auditable, and explainable classification at scale.

Due to the ASA-PS classification’s basis on subjective clinical judgment, the ideal state for any model to be measured against is itself imperfect. Rather than targeting perfect agreement with reference labels, a more meaningful ideal for an automated classification system would be ±1-class accuracy approaching 100% with a macro-*F*_1_-score approaching, though not necessarily exceeding, the expert panel’s benchmark. The parallel ensemble’s exact match accuracy of 63% (95% CI 0.580‐0.670) on the held-out test set aligns with the documented limitations of ASA-PS classification itself. Our model’s performance demonstrates effective extraction of signal from inherently subjective clinical data, approaching the limits imposed by the classification system’s fundamental ambiguity.

The parallel ensemble model’s 98.0% accuracy within one ASA-PS class (95% CI 0.968‐0.992) on the held-out test set serves as a critical safety mechanism. Extreme misclassifications (errors of 2 or more classes) in perioperative medicine pose a great risk to patient safety, potentially routing high-acuity patients to inadequate care before procedures or inadequately resourced surgical venues. By heavily reducing these outliers, the parallel ensemble ensures automated classifications remain within clinically acceptable bounds. While exact match accuracy remains the ideal, this containment of extreme errors provides the operational stability necessary for safe implementation across diverse perioperative settings.

The clinical consequences of misclassification are not uniform across the ASA-PS rank order. Errors at the lower end, between ASA-PS I and II, carry minimal operational consequence, as both classes represent patients appropriate for ambulatory and outpatient settings with equivalent resource requirements. As classification rank increases, the stakes escalate: a one-class error at the ASA-PS II-III boundary carries meaningful downstream consequences because many ambulatory surgery centers and office-based facilities use ASA-PS thresholds as explicit eligibility criteria, so an under-classified ASA-PS III patient may be routed to a facility unprepared for their acuity, while an over-classified patient may unnecessarily consume high-acuity resources. ASA-PS is, however, only 1 determinant of venue: procedural risk should codetermine where surgery occurs, and an ASA-PS III patient whose comorbidities require no additional clinical support may safely undergo a low-risk procedure such as cataract surgery in an ambulatory surgery center [30]. Preoperative evaluation and testing should likewise be matched to the surgical task at hand, an alignment identified as a primary source of net cost reduction in the perioperative surgical home model [31]. By effectively eliminating errors of 2 or more classes, the parallel ensemble prevents the most consequential misclassifications, those that would route high-acuity patients into fundamentally inadequate care environments.

This work builds upon and advances multiple approaches to automated ASA-PS classification. Prior efforts have explored 3 primary strategies: structured data-only models, text-only models, and other advanced approaches.

Text-based and retrieval-augmented approaches provide the closest comparators. Chung et al [16] evaluated multiple natural language processing models on preoperative notes from 19,740 patients, with fastText (Facebook AI Research [FAIR], Meta Platforms Inc.) achieving an accuracy of 0.617 and BioClinicalBERT 0.584; Yoon et al [32] ClinicalBigBird achieved an accuracy of 0.728, outperforming board-certified anesthesiologists in specificity, precision, and *F*_1_, though within a single South Korean health system whose highly standardized documentation and uniform data structures likely facilitated stronger model performance; and Chen et al [11] pioneered a retrieval-augmented generation (RAG) approach, using GPT-4 with retrieved similar cases to achieve 74.09% accuracy. Our parallel ensemble improved upon Wongtangman’s structured-data approach [13] despite using a similar feature set, likely attributable to our integration of LLM-extracted diagnoses from unstructured clinical text.

Our parallel ensemble’s macro-*F*_1_-score of 0.530 (95% CI 0.350‐0.600), exact match accuracy of 0.630 (95% CI 0.580‐0.670), and ±1-class accuracy of 0.980 (95% CI 0.968‐0.992) advance these efforts in 3 key ways. First, no prior team has used a parallel ML-LLM architecture for ASA-PS prediction. Second, while Chen et al [11] RAG approach achieved slightly higher accuracy (74.09% vs 63%), our model provides structured explanations without requiring a retrieval database, making it more deployable in resource-constrained settings. Finally, our performance metrics approach the theoretical ceiling (*F*_1_-score=0.59) established by expert macro-*F*_1_-score against the clinical reference standard while maintaining safety through near-perfect extreme error prevention, a metric not reported in prior studies but critical for clinical deployment. The consistent finding across all studies, including ours, is that neither structured nor unstructured data alone maximizes performance. Our contribution demonstrates that parallel architecture with transparent weighting offers the most promising path toward clinically deployable ASA-PS automation.

### Limitations

This study has limitations that warrant consideration. First, as with all research involving the ASA-PS system, the absence of a verified gold standard remains a fundamental challenge. Because ASA-PS assignment is inherently subjective, the model is trained to replicate human behavior rather than an absolute physiological truth. Consequently, perfect alignment between the model and provider is neither possible nor desirable. While the ideal ground truth would be a massive hand-labeled dataset derived from an expert panel of anesthesiologists, the thousands of encounters required for robust model training make such a dataset difficult and time-intensive to obtain.

Second, the LLM-generated explanations (Textbox 2) represent a proof-of-concept rather than production-ready outputs. While these explanations are not likely to satisfy every anesthesia provider, they demonstrate a critical principle: transparent algorithmic reasoning is essential for clinical adoption. Anesthesiologists will not trust black-box ASA-PS predictions; they require visibility into the model’s decision-making process to validate or challenge automated assessments. These preliminary outputs show that the model considers clinically relevant factors (comorbidities, functional status, and treatment burden) aligned with how providers conceptualize ASA-PS scoring. Though requiring substantial refinement before deployment, this transparency framework illustrates how AI could serve as a cognitive multiplier in resource-constrained preoperative clinics by helping triage thousands of patients, flagging complex cases for intensive review while streamlining straightforward evaluations. By making reasoning explicit, we establish the foundation of trust necessary to realize efficiency gains without compromising care quality.

Lastly, this study was conducted at a single institution with 3000 encounters in which ASA-PS V cases were extremely rare. Also, race and ethnicity data were not available and therefore could not be evaluated. Race and ethnicity fields are not captured within the structured-data pipeline used for this study, so the fairness audit was necessarily limited to language, gender, and age subgroups. Because race and ethnicity are well-documented determinants of documentation patterns and electronic health record data quality [33], their absence meaningfully constrains the comprehensiveness of the fairness evaluation, and prospective validation will prioritize their inclusion. A post hoc subgroup fairness audit on the held-out test set identified a substantial performance gap for Spanish-speaking patients (n=13; macro-*F*_1_=0.216; accuracy=0.385), corresponding to deficits of 31.4 and 24.5 percentage points below the overall benchmark, respectively. This likely reflects the predominance of English-language clinical documentation in both the source data and the LLM training corpus. This deficit should be interpreted with caution: computing macro-*F*_1_-score across 5 classes on 13 observations is mathematically unstable, and the figure reflects a directional signal warranting further investigation rather than a precise quantitative estimate. Performance for gender subgroups was comparable to the overall benchmark. Age-subgroup fairness analysis was constrained by missing data in a subset of encounters and should be interpreted with corresponding caution. These equity findings warrant careful consideration before deployment in multilingual or demographically diverse settings, and prospective multilingual validation is a priority for future work. Additionally, the structured data inputs rely in part on a proprietary LLM-based diagnosis extraction pipeline, which was internally validated at a sensitivity of 0.738 (95% CI 0.719‐0.752), precision of 0.845 (95% CI 0.832‐0.857), and specificity of 0.978 (95% CI 0.976‐0.980), and extraction errors may propagate to the XGBoost component. XGBoost output probabilities were not formally calibrated prior to applying the confidence gating threshold in the sequential hybrid architectures; formal calibration using out-of-fold training predictions is recommended before clinical deployment. Within the parallel ensemble specifically, final predictions average the uncalibrated XGBoost probability distribution with the GPT-4o output. Several factors limit the practical impact of this miscalibration: the GPT-4o component contributes a hard classification encoded as a one-hot vector rather than a competing probability distribution, the XGBoost stream carries only 30% of the weighted prediction (alpha=.30), and macro-*F*_1_-score is stable across alpha=.20‐.45, indicating low sensitivity to the precise XGBoost probabilities. Formal calibration via isotonic regression or Platt scaling on out-of-fold training predictions nonetheless remains preferable and should precede prospective deployment. These factors, together with the single-center design, constrain the generalizability of our findings. The 0.04 macro-*F*_1_-score delta between validation (0.574) and held-out test (0.530) performance reflects the expected consequence of selecting the ensemble weight on a finite validation set, inherent to any train, validation, and test design; alpha was fixed at 0.30 prior to test-set evaluation with no further tuning. Overlapping bootstrap CI across both estimates suggest this gap reflects sampling variance rather than substantial overfitting, though evaluation on larger independent cohorts is needed to confirm generalizability.

The exclusion of emergency cases constrains operational scope, though deliberately so: true emergencies lack the complete preoperative documentation both pipelines require, and the emergency modifier reflects urgency rather than the comorbidity burden the model predicts. In Kahneman [34] terms, emergency care demands fast thinking, and in such rapidly evolving clinical contexts an automated baseline classifier becomes irrelevant. The framework is intended for the deliberative, slow-thinking setting of elective and semielective pathways, where documentation is complete and consistent baseline classification has operational consequences. Future research involving multicenter datasets, a broader ASA-PS class distribution, and prospective external validation is necessary to establish the robustness of this parallel ensemble framework across diverse perioperative environments.

ASA-PS classification, despite its limitations, remains integral to perioperative workflows, and reducing classification variability through standardized algorithms addresses a persistent operational challenge without supplanting clinical judgment. Ferrari et al [12] found that anesthesiologists agreed with algorithmic ASA-PS predictions in 90.7% of cases, suggesting that providers will accept automated classification, but that acceptance is conditional on the transparency requirement described above: anesthesiologists will not trust black-box predictions, and it is precisely this need that the explanation component of our framework is designed to meet.

The potential impact is greatest given current financial and workforce headwinds. Persistent shortages of nurses [35], anesthesiologists [36], and perioperative personnel [37] have strained preoperative evaluation capacity while surgical volumes [38] continue rising alongside an aging population with greater medical complexity [39]. These converging pressures demand scalable solutions that maintain quality while expanding access.

### Conclusions

We present a parallel ML-LLM ensemble built from a single center that achieved 63% (95% CI 0.580‐0.670) exact match accuracy and macro-*F*_1_-score of 0.53 (95% CI 0.35‐0.60) on a true held-out test set for ASA-PS classification, with 98% (95% CI 0.968‐0.992)±1-class accuracy, approaching the theoretical performance ceiling established through independent review by 2 board-certified anesthesiologists (AF and GF). This architecture represents the first parallel hybrid approach to ASA-PS prediction, demonstrating that independent processing of structured and unstructured data before weighted integration constitutes a dual-input model rather than a purely ML or LLM approach. By providing transparent explanations alongside predictions, this framework represents not merely a technical advance but a practical response to the evolving demands of perioperative medicine, with the potential to improve patient safety, operational efficiency, and access to care across diverse health care settings.

## Supplementary material

10.2196/90724 Multimedia Appendix 1 Parallel ensemble alpha sweep results on the validation set and per-class Shapely additive explanations (SHAP) summary plots for the Extreme Gradient Boosting (XGBoost) component.

## Abbreviations

ASA-PS: American Society of Anesthesiologists-Physical Status
ClinicalBERT: Clinical Bidirectional Encoder Representations from Transformers
FAIR: Facebook AI Research
GPT: Generative Pretrained Transformer
HIPAA: Health Insurance Portability and Accountability Act
*ICD-10*: *International Statistical Classification of Diseases, Tenth Revision*
IRB: Institutional Review Board
LightGBM: Light Gradient Boosting Machine
LLM: large language model
ML: machine learning
RAG: retrieval-augmented generation
SHAP: Shapley additive explanations
TRIPOD-AI: Transparent Reporting of a Multivariable Prediction Model for Individual Prognosis or Diagnosis-AI
UAMS: University of Arkansas for Medical Sciences
XGBoost: Extreme Gradient Boosting
