# Supplementary material for: American Society of Anesthesiologists Physical Status Classification: Hybrid Machine Learning–Large Language Model Ensemble Retrospective Validation Study
Source: J Med Internet Res. 2026 Nov 5;28:e90724. doi: 10.2196/90724 (PMC13739266; doi:10.2196/90724)
Supplement: Supplementary file 1 [file jmir-v28-e90724-s001.docx]

Appendix Table 1: Parallel Ensemble Alpha Sweep Analysis with exact match accuracy, macro-F1, and Exact Match ±1-Class Accuracy

| α | Macro-F1 | 95% CI (F1) | Exact Match Accuracy | 95% CI (Accuracy) | ±1-Class Accuracy | 95% CI (±1-Class) |
| --- | --- | --- | --- | --- | --- | --- |
| 0.00 | 0.642 | [0.525-0.691] | 0.668 | [0.628, 0.708] | 0.982 | [0.970-0.992] |
| 0.05 | 0.642 | [0.522-0.690] | 0.668 | [0.629, 0.709] | 0.982 | [0.970-0.992] |
| 0.10 | 0.642 | [0.522-0.690] | 0.668 | [0.629, 0.709] | 0.982 | [0.970-0.992] |
| 0.15 | 0.642 | [0.523-0.691] | 0.668 | [0.629, 0.709] | 0.982 | [0.970-0.992] |
| 0.20 | 0.574 | [0.418-0.662] | 0.668 | [0.625, 0.708] | 0.982 | [0.970-0.992] |
| 0.25 | 0.576 | [0.400-0.669] | 0.668 | [0.623, 0.709] | 0.982 | [0.970-0.992] |
| 0.30 ^a^ | 0.574 | [0.404-0.671] | 0.666 | [0.622, 0.710] | 0.982 | [0.970-0.992] |
| 0.35 | 0.576 | [0.406-0.672] | 0.666 | [0.623, 0.710] | 0.984 | [0.971-0.993] |
| 0.40 | 0.576 | [0.404-0.671] | 0.666 | [0.623, 0.710] | 0.984 | [0.971-0.993] |
| 0.45 | 0.576 | [0.405-0.671] | 0.666 | [0.623, 0.710] | 0.984 | [0.971-0.993] |
| 0.50 | 0.469 | [0.404-0.616] | 0.598 | [0.552, 0.638] | 0.968 | [0.952-0.982] |
| 0.55 | 0.409 | [0.360-0.539] | 0.592 | [0.550, 0.636] | 0.962 | [0.944-0.978] |
| 0.60 | 0.409 | [0.358-0.541] | 0.592 | [0.546, 0.636] | 0.962 | [0.946-0.978] |
| 0.65 | 0.409 | [0.355-0.528] | 0.592 | [0.546, 0.636] | 0.962 | [0.942-0.978] |
| 0.70 | 0.409 | [0.357-0.538] | 0.592 | [0.548, 0.632] | 0.962 | [0.946-0.978] |
| 0.75 | 0.419 | [0.365-0.550] | 0.600 | [0.556, 0.642] | 0.962 | [0.946-0.978] |
| 0.80 | 0.377 | [0.332-0.494] | 0.546 | [0.502, 0.588] | 0.794 | [0.756-0.830] |
| 0.85 | 0.382 | [0.340-0.503] | 0.546 | [0.502, 0.588] | 0.782 | [0.746-0.818] |
| 0.90 | 0.382 | [0.339-0.505] | 0.546 | [0.502, 0.588] | 0.782 | [0.744-0.818] |
| 0.95 | 0.382 | [0.335-0.512] | 0.546 | [0.504, 0.588] | 0.782 | [0.746-0.816] |
| 1.00 | 0.333 | [0.288-0.388] | 0.662 | [0.622, 0.706] | 0.990 | [0.982-0.998] |

Caption: Systematic evaluation of the parallel ensemble model performance across 21 different alpha (α) values on the validation set (n=500), where α represents the relative weighting between machine learning and large language model predictions (α=1.0 indicates 100% ML weight; α=0.0 indicates 100% LLM weight). Macro-F1 remained stable at 0.574-0.576 across α = 0.25–0.45, demonstrating that the selection of α=0.30 is robust and not sensitive to the precise weighting value. Final model performance was evaluated on a held-out test set not used during model development or hyperparameter selection.

^a^ Selected alpha. α = weight assigned to XGBoost (ML); 1−α = weight assigned to GPT-4o (LLM). Bootstrap 95% CIs based on 1,000 resamples on the validation set (n=500).

An α=0.30 was chosen as a stable inflection point that balances LLM-driven gains in label diversity (F1) with high agreement to ground truth (accuracy), while remaining aligned with the theoretical upper bound of achievable model performance. On the validation set, this configuration achieved a macro-F1 of 0.574 (95% CI [0.404-0.671]), exact match accuracy of 0.666 (95% CI [0.622-0.710]), and within-one-class accuracy of 0.982 (95% CI [0.970-0.992]).

Appendix Figure 1: SHAP summary(beeswarm) plots

Caption: SHAP summary (beeswarm) plots showing how individual structured features contribute to each ASA-PS class prediction (ASA-PS I–V) for the XGBoost component of the parallel ensemble. Each point represents one surgical encounter its position on the x-axis is the SHAP value, the feature's contribution to that class's predicted score, with positive values pushing the prediction toward the labeled ASA class and negative values pushing away. Point color is mapped from untransformed feature values (blue = low, red = high). Gray points arise from two sources: binary flags and categorical variables (for example, is_prisoner, case_service, vasopressin_flag) are non-numeric in untransformed form and are rendered gray for every observation regardless of missingness, whereas gray points on continuous features denote values missing prior to imputation. SHAP values were computed on the preprocessed feature matrix supplied to the model, in which numeric missingness was mean-imputed and categorical missingness was retained as an explicit level. Within each panel, features are ordered by mean absolute SHAP value. These attributions reflect the structured-data (XGBoost) stream only; the large language model component contributes the complementary natural-language rationale shown in Table 5. Attributions for the well-populated ASA II and III classes are dense and stable, whereas ASA V attributions rest on few true ASA V examples and should be interpreted with caution, reflecting general acuity proxies (age, medication burden) rather than definitive organ-failure markers
